# Successful treatment of early allograft dysfunction with cinacalcet in a patient with nephrocalcinosis caused by severe hyperparathyroidism: a case report

**DOI:** 10.1186/s13104-017-2477-0

**Published:** 2017-04-08

**Authors:** Boonyarit Cheunsuchon, Suchai Sritippayawan

**Affiliations:** 1 Department of Pathology, Faculty of Medicine Siriraj Hospital, Mahidol University, 2 Prannok Road, Bangkok, 10700 Thailand; 2 Division of Nephrology, Department of Medicine, Faculty of Medicine Siriraj Hospital, Mahidol University, Bangkok, Thailand

**Keywords:** Cinacalcet, Nephrocalcinosis, Early allograft dysfunction, Case report, Hyperparathyroidism

## Abstract

**Background:**

Hyperparathyroidism is common in patients undergoing kidney transplantation. Occasionally, this condition can cause early allograft dysfunction by inducing calcium phosphate deposition in the allograft, which results in nephrocalcinosis. Although nephrocalcinosis occurs occasionally in kidney allografts, it has only rarely been reported in the literature.

**Case presentation:**

Here, we present the case of a 58-year-old Thai woman with severe hyperparathyroidism who received a living-related kidney transplant from her 35-year-old son. Our patient developed allograft dysfunction on day 2 post-transplantation despite good functioning graft on day 1. Allograft biopsy showed extensive calcium phosphate deposition in distal tubules. She was treated with cinacalcet (a calcimimetic agent) and aluminum hydroxide. Allograft function was restored to normal within 1 week after transplantation with greatly reduced intact parathyroid hormone level.

**Conclusion:**

Hyperparathyroidism in early functioning allograft causes elevated calcium and phosphate concentration in distal tubules resulting in nephrocalcinosis. The massive calcium phosphate precipitation obstructs tubular lumens, which leads to acute tubular dysfunction. Treatment of nephrocalcinosis with cinacalcet is safe and may improve this condition by increasing serum phosphate and reducing serum calcium and intact parathyroid hormone.

## Background

Advances in tissue typing, crossmatching techniques, and immunosuppression have significantly reduced the risk of early renal allograft dysfunction due to immunological injury [1]. Although short-term graft survival is markedly improved, long-term graft outcome remains uncertain [1]. Recently, many studies have demonstrated the importance of nonimmunological processes (e.g., drug toxicity, infections, and recurrent/de novo glomerular diseases) that can cause allograft deterioration [2]. Nephrocalcinosis is a common finding in renal allografts, with an incidence of 80% at 10 years after transplantation [3]. However, nephrocalcinosis is rarely recognized as the cause of early allograft dysfunction [4]. Here, we present a histological finding of nephrocalcinosis in a renal allograft of a patient with early graft dysfunction. Clinical findings, possible etiologies, pathogenesis, and treatment are all briefly discussed.

## Case presentation

**Fig. 1:**
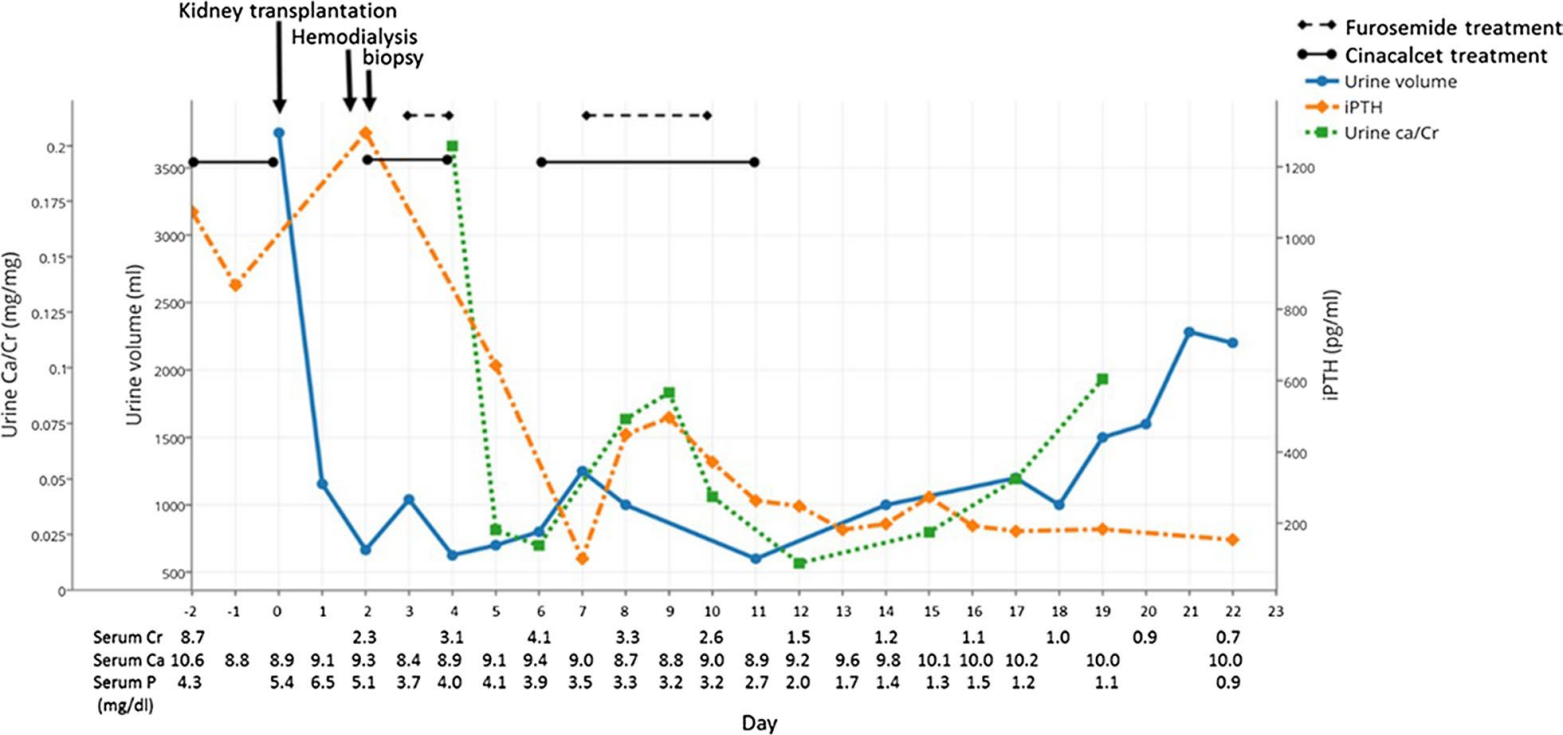
Clinical course and laboratory findings before and after kidney transplantation. The *solid and dash black lines* demonstrate the duration of cinacalcet (25 mg/day) and furosemide (250–1000 mg/day) treatments, respectively

A 58-year-old Thai woman developed end-stage renal disease from lupus nephritis and had been on hemodialysis for 2 years. She had markedly elevated serum intact parathyroid hormone (iPTH) of 113.8 pmol/L (1.6–6.9 pmol/L), increased serum calcium (Ca) of 2.65 mmol/L (2.10–2.37 mmol/L), and a phosphorus (P) level of 1.39 mmol/L (1.13–1.78 mmol/L). She was on Lanthanum carbonate, later converted to aluminum hydroxide, but was not on 1 alpha cholecalciferol, vitamin D analogs or calcium supplements. Daily urine output was 130 mL and her urine pH was high at 7.5 without urinary tract infection or sodium bicarbonate use. On 26 March 2014, our patient received a renal transplant from her haplo-identical and compatible blood group 35-year-old son. The radionuclide renogram demonstrated normal function of both of the donor’s kidneys. Pretransplant luminex crossmatch and panel reactive antibody tests were negative. Parathyroid sestamibi scan was not done because the doctor and patient did not want to do a parathyroidectomy. Cinacalcet (50 mg, once a day), a calcimimetic agent, and aluminum hydroxide (Al(OH)_3_) (1000 mg, three times a day) were prescribed for 2 days prior to transplantation. Serum Ca, P, and iPTH were decreased to 2.20, 1.74 mmol/L, and 92 pmol/L at the time of transplantation, respectively (Fig. 1). Induction therapy consisted of tacrolimus, mycophenolate mofetil, and steroid without interleukin-2 receptor antagonists as indicated in institutional protocol.

**Fig. 2:**
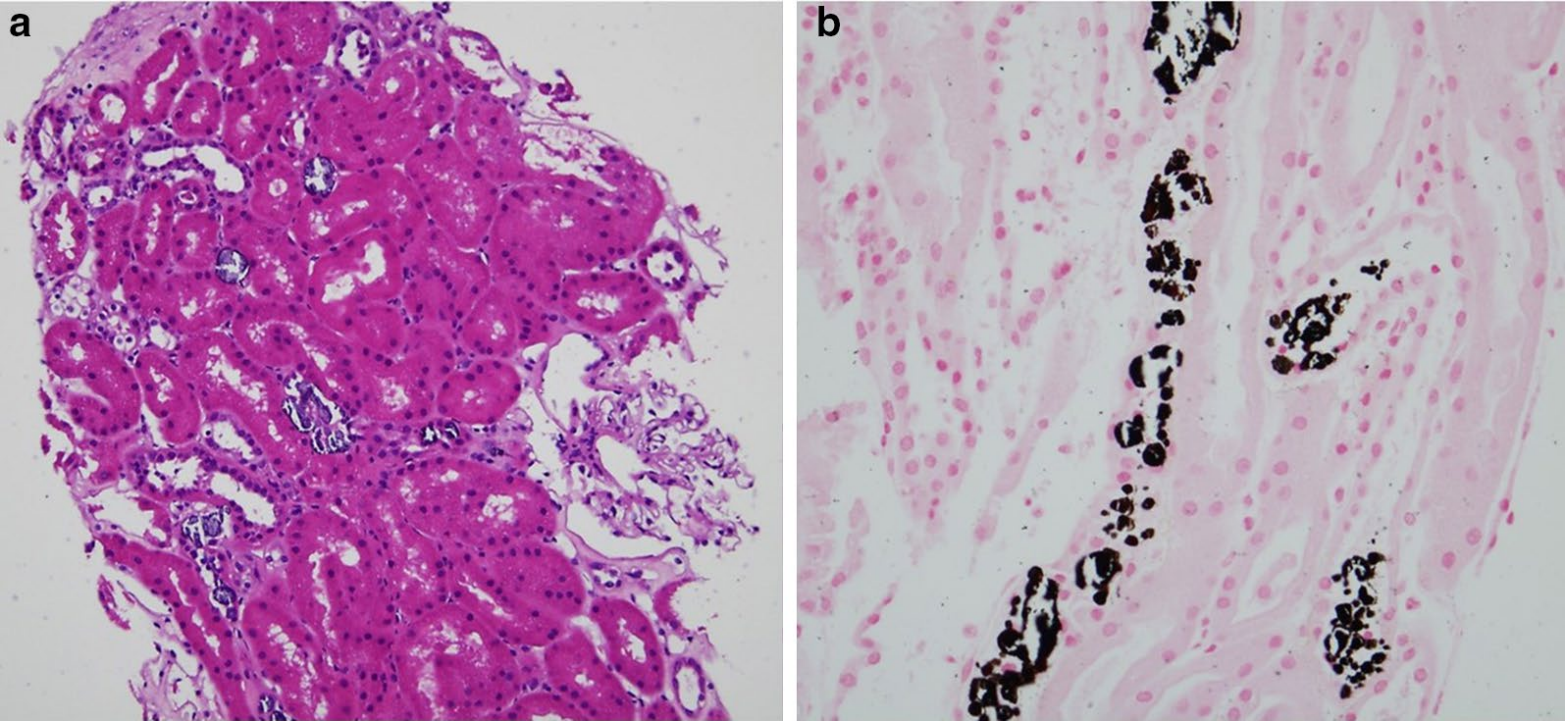
**a** Numerous intratubular calcium crystals are noted in the tubular lumens. The absence of interstitial inflammatory cell infiltration rules out acute T cell-mediated rejection (H&E stain, original magnification ×200). **b** Presence of calcium phosphate crystals proven by von Kossa staining (von Kossa stain ×400)

The patient had immediate graft function with 3000 mL of urine during day 1 after transplantation. However, urine output decreased to 1150 mL on day 2, with serum Ca, P, and iPTH levels increased to 2.33 mmol/L, 1.65 mmol/L, and 137.4 pmol/L, respectively (Fig. 1). Serum creatinine (SCr) was significantly elevated to 280 µmol/L on day 4. Doppler ultrasound of the transplanted kidney showed no hydronephrosis or vessel-related problems. Cinacalcet (25 mg) and Al(OH)_3_ were restarted again on day 2. Intravenous furosemide was also prescribed to enhance urine output. Tacrolimus level were 6.5–11.9 ng/mL. Hemodialysis was started and allograft biopsy was performed on day 2 after transplantation. The graft biopsy contained 25 glomeruli with no presence of glomerulitis or fibrin thrombi. There were, however, more than 20 foci of intratubular basophilic crystals (Fig. 2a). The crystals were positive for von Kossa stain, indicating calcium phosphate precipitation (Fig. 2b). Under polarized light, crystals showed no birefringence that is characteristic of calcium oxalate. Interestingly, all of the crystals were located in the distal tubules. There was no evidence of tubular injury or rejection. Immunofluorescence studies for IgG, IgA, IgM, C3, C1q, kappa, lambda, fibrinogen, and C4d were all negative. The histopathological diagnosis was nephrocalcinosis caused by intratubular precipitation of calcium phosphate crystals, most likely due to severe hyperparathyroidism. Urine pH before and 1 day after transplantation was 7.5 and 6.0, respectively. Our patient did not use any phosphate-containing laxatives. We withheld the cinacalcet for 24 h due to a high urine calcium/creatinine (Ca/Cr) ratio (0.57 mmol/mmol), but restarted later after the ratio was decreased to 0.08 and continued for 1 week without any change in immunosuppressive agents. SCr and iPTH declined to less than 176.8 µmol/L and 21.2 pmol/L, respectively, while serum Ca, P, and urine pH were maintained between 2.10–2.25 mg/dL, 0.87–1.32 mmol/L, and 5.5–6.0, respectively. Only one hemodialysis treatment was required.

At 2 years after transplantation, our patient has slightly high serum iPTH (13.8–21.2 pmol/L) and Ca (2.65 mmol/L) levels, with low level of serum P (0.84 mmol/L). Current immunosuppressive agents include tacrolimus, mycophenolate mofetil, and prednisolone.

## Discussion

Nephrocalcinosis can be found as early as 6 weeks (6% in protocol biopsies) after transplantation [5]. The incidence progressively increases to almost 80% by 10 years after transplantation [3]. The presence of nephrocalcinosis in renal allografts was found to be associated with chronic allograft nephropathy [5]. Although nephrocalcinosis effect was not significant in the short-term, the long-term consequences were unclear [6]. A possible etiology of nephrocalcinosis in this patient may have been metabolic derangement associated with severe hyperparathyroidism. Nephrocalcinosis was more frequently seen in allograft patients with hyperparathyroidism than in those without this condition [5, 6]. Hyperparathyroidism increases the filtered load of calcium from high serum calcium level and decreases phosphate reabsorption in proximal tubules, both of which elevate calcium and phosphate concentrations in distal tubules. Our patient also had high urine pH (7.5) before transplantation, which enhanced calcium phosphate precipitation. Unfortunately, we have no data on urine citrate, ammonium, and sulfate levels, which might help to indicate the cause of the high urine pH in this patient. Calcium phosphate precipitation also causes tubular cell injury, which causes the distal tubules to express proteins, such as osteopontin and hyaluronan. These molecules promote calcium phosphate adhesion to the surface of tubular cells [7]. Massive calcium phosphate precipitation causes tubular obstruction, which leads to acute allograft dysfunction.

Calcineurin inhibitors were associated with nephrocalcinosis in renal allografts. However, the incidence was decreased in later study and found not to be different from calcification due to acute tubular necrosis [8]. This was probably the result of reduced dose of immunosuppressive agents used in current practice.

Hyperparathyroidism is common in patients undergoing renal transplantation. Association has been established between hyperparathyroidism and delayed graft function [9]. Although some reports appear in the literature [4, 10–13], all of the reports are single-patient case reports (none are large case series) of patients with early graft dysfunction and nephrocalcinosis (Table 1). Among those patients, nephrocalcinosis was detected as early as 5 days after transplantation. Four of 5 cases showed concurrent lesions (3 acute tubular necrosis, 1 borderline rejection). Serum calcium and phosphate levels varied from normal to markedly increased. Two patients had improved graft function after supportive treatment and two others required parathyroidectomy. Only one patient had graft failure, which was found to be caused by gradual increase in calcium crystal deposition in the follow-up biopsy.

**Table 1:** Case reports of early allograft dysfunction due to nephrocalcinosis

	Iguchi et al.	Sewpaul et al.	Backman et al.	Wong et al.	Manfro et al.
Patient gender/age (year)	Female/56	Male/48	Male/36	Female/44	Male/36
Allograft type	Living-related	Living-related	Living	Cadaveric	Cadaveric
Biopsy time after transplant w/nephrocalcinosis	10 days	7 days	3 days	18 days (biopsy at 3 days showed acute cellular rejection w/o calcium crystals)	15 days
Other lesions	Borderline acute rejection	Acute tubular necrosis	None	Acute tubular necrosis	Acute tubular necrosis
Serum calcium	Slightly increased	Markedly increased	Normal	Normal	Normal
Serum phosphate	Slightly increased	Slightly increased	Normal	Markedly increased	Markedly increased
Follow-up	Graft function improved after supportive treatment	Parathyroidectomy improved hypercalcemia and graft function	Parathyroidectomy improved graft function. Decreased amount of calcium crystals in follow-up biopsy	Graft loss within 5 months. Serial biopsy showed gradual increase in calcium crystals and tubulointerstitial fibrosis	Repeat biopsy at day 85 showed no calcification. Stable graft function at 1 year

Cinacalcet is a novel phenylalkylamine type II calcimimetic agent that allosterically modulates the calcium sensing receptor by increasing the sensitivity of the receptor to extracellular calcium, which leads to suppression of PTH transcription, secretion, and parathyroid gland hyperplasia. Activation of the calcium sensing receptor inhibits phosphate reabsorption at the proximal tubule and sodium chloride reabsorption at the thick ascending limb of Henle loop, which promotes calciuria as a result of the reduction in passive paracellular calcium reabsorption. Cinacalcet was approved by the United States Food and Drug Administration for treatment of parathyroid cancer and hyperparathyroidism in end-stage renal disease patients [14]. The benefit of a calcimimetic agent in post-renal transplant hyperparathyroidism was demonstrated in a randomized controlled trial [15]. In that study, cinacalcet demonstrated an ability to significantly increase serum phosphorus level and decrease serum calcium and iPTH over the study’s 52-week duration. In contrast to results from other case reports and retrospective studies, three studies in nephrocalcinosis that developed in transplanted kidneys found that cinacalcet did not significantly decrease glomerular filtration rate or increase urinary calcium excretion [16–18]. To date, no strong evidence has been presented that suggests that cinacalcet worsens kidney graft function or that it is associated with other severe adverse effects after transplantation. Urgent parathyroidectomy was recommended by a previous case report to improve graft function in a poorly-controlled hyperparathyroidism patient who experienced graft dysfunction due to acute tubular necrosis associated with intratubular calcification [12]. The use of calcimimetic agent in this condition has not been previously reported. We demonstrated the efficacy of cinacalcet in decreasing serum intact PTH level and calcium level and improving graft function in a patient with poorly-controlled severe hyperparathyroidism who developed early graft dysfunction from intratubular calcium deposition immediately after transplantation. Immediate graft function in a setting of high urine pH and hyperparathyroidism-associated hypercalciuria and hyperphosphaturia may have caused calcium phosphate crystallization in this patient. During acute kidney injury, urine Ca/Cr ratio was found to be associated with furosemide and cinacalcet therapy. Whether the increase in the urine Ca/Cr ratio was due to high urine calcium or low urine creatinine has yet to be established. In any case, our patient’s kidney function was improved within one week after starting cinacalcet treatment, with accompanying decreases in serum calcium and iPTH. At time of discharge, our patient had good kidney function (SCr 67.2 mg/dL), but still had a slightly elevated iPTH that ranged from 15.9 to 21.2 ng/L.

## Conclusion

Severe hyperparathyroidism can cause early allograft dysfunction from intratubular calcium crystal obstruction after kidney transplantation. Perioperative treatment with cinacalcet was safe and effective in reversing the effects of nephrocalcinosis and improving kidney function in this patient.

## Abbreviations

Al(OH)_3_: aluminum hydroxide
Ca: calcium
iPTH: intact parathyroid hormone
P: phosphorus
SCr: serum creatinine
